# Diagnostic Dilemma of a Giant Paratubal Cyst in an Adolescent Female Mimicking a Mesenteric Cyst: A Case Report

**DOI:** 10.7759/cureus.108621

**Published:** 2026-05-11

**Authors:** Yahia Y Akeely, Saleh A Alesa, Swaid Saulat, Hussam H Farrash, Louai M Alahdal

**Affiliations:** 1 Emergency Department, Security Forces Hospital Program, General Directorate of Medical Services, Ministry of Interior, Riyadh, SAU

**Keywords:** adnexal cyst, diagnostic dilemma, giant cyst, mesentric cyst, paratubal cyst

## Abstract

Paratubal cysts are rare adnexal masses that arise from the mesosalpinx and are typically small and asymptomatic. Giant paratubal cysts are a rare entity and a diagnostic challenge, especially in adolescents, due to nonspecific presentation and radiological similarity to other intra-abdominal cystic masses. A 15-year-old female presented with progressive abdominal distension and mild, nonspecific abdominal pain of three months’ duration, associated with recent menstrual irregularities. Physical exam showed a distended, non-tender abdomen. Imaging studies, including computed tomography (CT) and magnetic resonance imaging (MRI), revealed a huge cystic abdominopelvic lesion of around 30 cm displacing the adjacent loops of bowel and showing right-sided hydroureteronephrosis. The origin of the cyst was uncertain, and differential diagnoses were mesenteric cyst and adnexal cyst. Laboratory investigations, including tumor markers, were within normal limits. Laparoscopic exploration was carried out, and a large cyst arising from the left fallopian tube was seen. Approximately 4.6 L of clear fluid was aspirated, followed by complete cyst excision with left salpingectomy. Histopathological examination showed a benign serous cystadenoma with no evidence of malignancy. The patient was well in the follow-up and postoperative period. Giant paratubal cysts are uncommon in the adolescent population and are often misdiagnosed preoperatively because imaging features overlap with ovarian and mesenteric cysts. Most are benign despite their size, but surgical excision is advised because of risks of complications such as torsion, rupture, and pressure effects on nearby organs. With adequate expertise, minimally invasive approaches can be safely performed with reduced morbidity and a faster recovery, even with such a large cyst.

## Introduction

Paratubal cysts are benign adnexal lesions arising from the mesosalpinx between the ovary and the fallopian tube. They constitute approximately 5%-20% of adnexal masses [1,2]. They are most frequently diagnosed in women of reproductive age, with only a small proportion of cases occurring in adolescents [3].

The majority of paratubal cysts are small and asymptomatic, with most measuring less than 10 cm in diameter. Giant paratubal cysts are rare and are usually defined as those measuring >15 to 20 cm [4,5]. Reported cases in pediatric and adolescent populations often present with nonspecific symptoms and diagnostic uncertainty [6,7].

Clinically, they induce nonspecific symptoms such as abdominal distension and vague abdominal pain, and when large, they may exert pressure on neighboring organs [8]. Paratubal cysts are frequently misdiagnosed by imaging studies as paraovarian, ovarian, or mesenteric cysts, making the preoperative diagnosis difficult [9,10].

Ultrasonography, computed tomography (CT), and magnetic resonance imaging (MRI) are employed in the evaluation of cystic masses, but determining the exact origin of large lesions remains challenging [8]. Surgical excision is the treatment of choice. There is a trend toward minimally invasive treatment modalities, especially for young patients, to preserve fertility [11-13].

This case is being reported to contribute to the limited literature on giant paratubal cysts in adolescents, particularly of extreme size, and to show that laparoscopic management can be performed safely and effectively even in difficult conditions.

## Case presentation

A 15-year-old girl with no significant past medical or surgical history presented with progressive abdominal distension and mild, nonspecific abdominal pain for the past three months. She also had a history of recent menstrual irregularities for the last two months. There was no history of fever, vomiting, or urinary symptoms.

On examination, the patient was hemodynamically stable with normal vital signs. Laboratory investigations revealed a hemoglobin level of 9.1 g/dL, a white blood cell count of 9.39 × 10⁹/L, and a platelet count of 521 × 10⁹/L. Electrolyte and renal function tests were within normal limits. Furthermore, all tumor markers, including beta-human chorionic gonadotropin (β-hCG), alpha-fetoprotein (AFP), CA-125, and CA 19-9, were within normal limits (Table 1). 

**Table 1:** Laboratory investigations on admission.

Test	Result	Normal reference range
Hemoglobin (Hb)	9.1 g/dL	12.0-16.0 g/dL
White blood cell count (WBC)	9.39 × 10⁹/L	4.0-11.0 × 10⁹/L
Platelet count	521 × 10⁹/L	150-400 × 10⁹/L
Sodium Na	138 mmol/L	135- 145 mmol/L
Creatinine	0.3 mg/dL	0.5-1.0 mg/dL
β-hCG	<1 IU/L	<1 IU/L (non-pregnant)
Alpha-fetoprotein (AFP)	2.65 ng/mL	<7.0 ng/mL
CA-125	30.80 U/mL	<35 U/mL
CA 19-9	2.0	<27 U/mL
Luteinizing hormone (LH )	5.10 IU/L	Follicular phase: up to 12.6 IU/L; midcycle: up to 95.6 IU/L; luteal phase: up to 11.4 IU/L
Estradiol	515.00 pmol/L	Follicular phase: 46-607 pmol/L; ovulation phase: 315-1,828 pmol/L; luteal phase: 161-774 pmol/L
Progesterone	21.70 nmol/L	Follicular phase: 0.6- 4.7 nmol/L; ovulation phase: 2.4-9.4 nmol/L; luteal phase: 5.3-86 nmol/L

**Figure 1:**
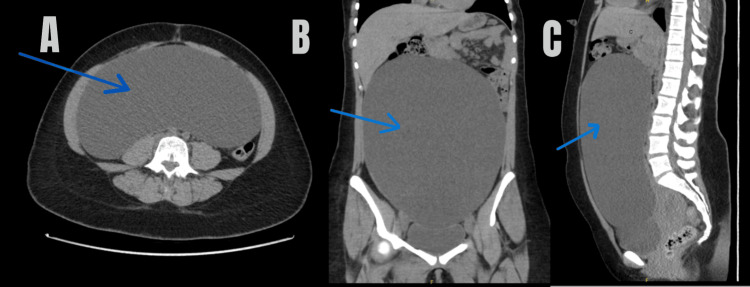
Computed tomography (CT) images demonstrating a giant abdominopelvic cyst. The blue arrow indicates the large cyst. (A) Axial (transverse) CT image showing a large, well-defined, homogenous cystic lesion occupying the majority of the abdominal cavity and causing significant displacement of adjacent bowel loops posteriorly. (B) Coronal CT image demonstrating the superior-inferior extent of the cyst, extending from the upper abdomen down to the pelvis, with marked mass effect on surrounding structures. (C) Sagittal CT image illustrating the full craniocaudal dimension of the cyst and its relationship to the pelvic organs, with inferior displacement of pelvic structures.

The initial non-contrast CT abdomen demonstrated a large, well-defined cystic lesion measuring approximately 27.3 cm in maximal dimension, occupying the abdominopelvic cavity. It caused mass effect on adjacent structures, including bowel loops and the right ureter, resulting in mild hydroureteronephrosis (Figure 1). The exact origin of the lesion was indeterminate. Differential diagnoses included an adnexal cyst and a mesenteric cyst.

**Figure 2:**
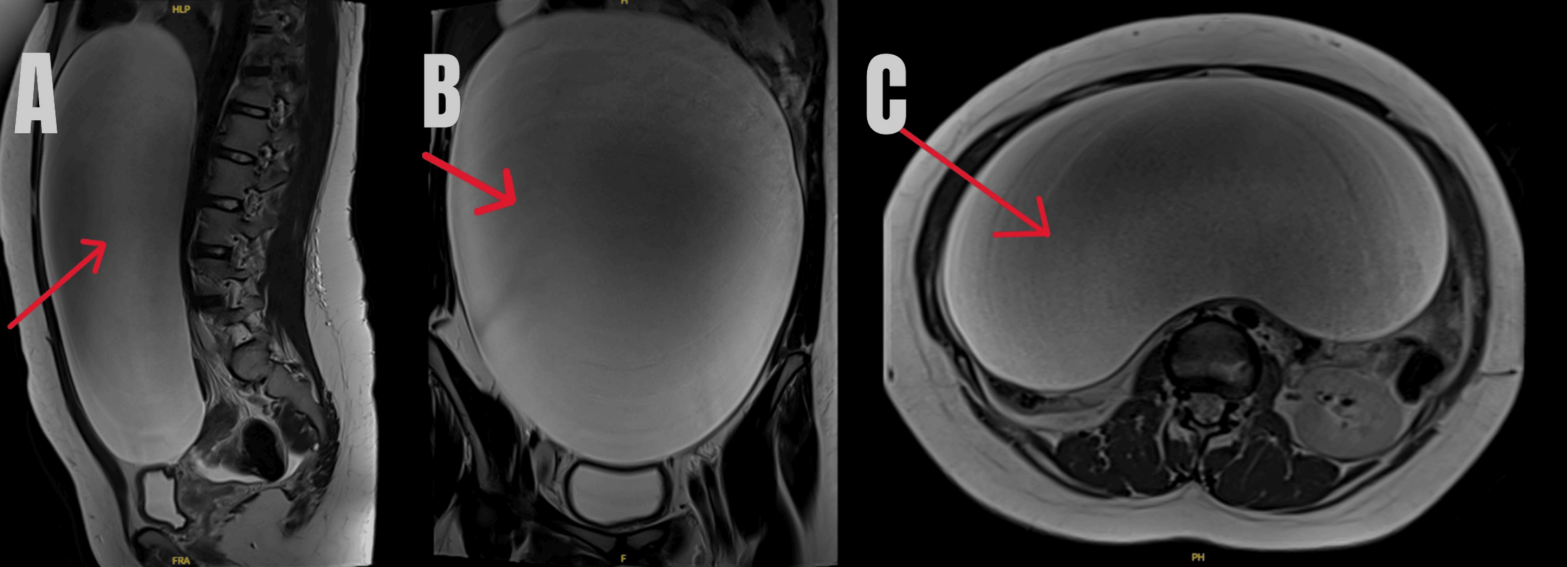
MRI of the abdomen and pelvis demonstrating a giant cystic lesion. The red arrow indicates the large cyst. (A) Sagittal T2-weighted MRI image showing a large, well-defined, homogeneously hyperintense cystic lesion occupying the abdominopelvic cavity, extending from the pelvis to the upper abdomen and causing posterior displacement of bowel loops and adjacent structures. (B) Coronal T2-weighted MRI image demonstrating the full craniocaudal extent of the cystic mass, with a smooth thin wall and no internal septations or solid components, exerting mass effect on surrounding abdominal organs. (C) Axial (transverse) T2-weighted MRI image showing a large, unilocular, hyperintense cyst with a thin wall, compressing adjacent bowel loops and retroperitoneal structures without evidence of invasion.

MRI of the abdomen and pelvis revealed a large, thin-walled, unilocular cystic lesion measuring approximately 28.7 × 22.2 × 9.4 cm, with homogeneous fluid signal and no solid components or septations (Figure 2). The left ovary was displaced medially and appeared separate from the lesion, favoring a paratubal or paraovarian cyst, although a mesenteric cyst could not be excluded.

The patient was discharged home and later returned for elective surgery after three weeks. Laparoscopic exploration in the operating room revealed a large cyst arising from the left fallopian tube, filling the entire abdominal and pelvic cavities. For visualization, controlled puncture and aspiration of approximately 4.6 L of clear fluid were undertaken intraoperatively. A left salpingectomy was performed with total cyst excision, as the fallopian tube was involved. The cyst measured 30 cm. Both the ovaries and uterus were normal.

The cyst was removed by endobag, and hemostasis was achieved. The postoperative course was uneventful, and she was discharged on the first postoperative day. Histopathology showed a benign serous cystadenoma without any signs of malignancy. The examination of the aspirated fluid by means of cytology was negative for malignant cells. The patient was asymptomatic and doing well at the three-week follow-up.

## Discussion

Giant paratubal cysts are uncommon, especially in adolescents, and only a small number of cases have been reported in the literature. As shown in Table 2, previously published cases vary in size, but most are smaller than the cyst observed in our patient. This comparison highlights that our case represents one of the largest reported to date, emphasizing its rarity and the importance of reporting such unusual presentations.

**Table 2:** Comparison of reported giant paratubal cysts in the literature and the present case. This table summarizes previously reported cases of giant paratubal cysts, including patient age, maximum cyst diameter, and type of surgical intervention, compared with the present case. Cyst size is reported as the largest recorded dimension.

Study (Author)	Age (years)	Size (cm)	Surgical approach	Histopathology
Tjokroprawiro [1]	30	22	Laparotomy	Benign cyst
Ooi et al. [3]	15	18	Laparotomy	Benign cyst
Romeo et al. [4]	15	26.7	Laparoscopy	Cystadenofibrosis
Zvizdic et al. [6]	15	25	Laparotomy	Serous cystadenoma
Almahmeed et al. [8]	26	34	Laparotomy	Benign cyst
Bhansakarya et al. [11]	25	27	Laparoscopy	Benign cyst
Archer et al. [13]	16	42	Mini laparotomy	Benign cyst
This case	15	30	Laparoscopy	Serous cystadenoma

As in our case, most paratubal cysts are misdiagnosed preoperatively because of their similarity to ovarian cysts, as reported by Tjokroprawiro [1]. Our patient had a large cystic lesion on both CT and MRI, with mesenteric and adnexal cysts being considered as alternative diagnoses. This finding was consistent with Almahmeed's findings, who also reported a case that resembled a mesenteric cyst [8].

Regarding size, our case (30 cm) is among the larger cysts reported in adolescents. Zvizdic reported a 25 cm cyst, while Romeo reported a 26.7 cm cyst [4,6]. Larger cysts (>30 cm) have been reported in adults, as described in Butureanu’s study [12].

The surgical approach has evolved from laparotomy to minimally invasive techniques. Traditionally, large cysts were managed with open surgery, as described by Vagholkar and Archer et al. [9,13]. However, more recent studies, such as those by Romeo et al. and Bhansakarya et al., have demonstrated successful laparoscopic management even for giant cysts [4,11]. Our case further supports this trend, as we performed controlled decompression followed by successful complete laparoscopic excision.

Fertility preservation is still an important consideration in adolescents. In most cases, the ovaries were preserved, and a few needed salpingectomy [4]. In our patient, a left salpingectomy was performed because the fallopian tube was completely involved; however, both ovaries were preserved, in accordance with current recommendations.

Most giant paratubal cysts are histopathologically benign. Stefanopol et al. reported that most are serous cystadenomas or simple cysts, with only a small percentage demonstrating borderline features [2]. Romeo and Zvizdic also reported benign histology in adolescent cases [4,6]. Our finding of a benign serous cystadenoma is consistent with the existing literature.

In conclusion, this case reinforces some important points: the diagnostic difficulty, the benign behavior of most lesions, and the feasibility of laparoscopic management even in giant cysts when performed by experienced surgeons.

## Conclusions

Giant paratubal cysts in adolescence are rare and may present with nonspecific symptoms, creating a diagnostic challenge. Large cystic abdominal or pelvic masses may be misdiagnosed on imaging, and a definitive diagnosis is often established intraoperatively.

The present case highlights the importance of considering paratubal cysts in the differential diagnosis of large abdominal/pelvic cystic lesions in adolescent patients. Although surgical management should be individualized based on patient characteristics, cyst features, intraoperative findings, and the surgeon’s expertise and experience, this case demonstrates that a minimally invasive laparoscopic approach can be a safe and feasible option, even in selected giant cysts, with favorable clinical outcomes. It should not be interpreted as a universal approach for all similar cases; rather, management must be tailored to each patient. Nevertheless, this case supports the growing evidence that less invasive techniques, such as laparoscopy, may successfully achieve effective treatment while minimizing surgical morbidity and preserving fertility when appropriately selected.

## References

[1] Tjokroprawiro BA (2021). Huge paratubal cyst: a case report and a literature review. Clin Med Insights Case Rep.

[2] Stefanopol IA, Baroiu L, Neagu AI (2022). Clinical, imaging, histological and surgical aspects regarding giant paraovarian cysts: a systematic review. Ther Clin Risk Manag.

[3] Ooi PY, Ahmad I, Azidah AK (2025). Navigating the diagnostic and management challenges of paratubal cysts in adolescents: a case report and primary care insights. Malays Fam Physician.

[4] Romeo P, Loria G, Martinelli C, Ercoli A, Romeo C (2022). Minimally invasive management of a giant paratubal cyst in an adolescent female: case report and review of the literature in the pediatric population. Front Pediatr.

[5] Woods GM, Kerlin BA, O'Brien SH, Bonny AE (2016). Giant paratubal cyst in adolescence: case report and minimally invasive technique. J Pediatr Adolesc Gynecol.

[6] Zvizdic Z, Bukvic M, Murtezic S, Skenderi F, Vranic S (2020). Giant paratubal serous cystadenoma in an adolescent female: case report and literature review. J Pediatr Adolesc Gynecol.

[7] Dechen S, Dorji N, Pradhan B (2025). A diagnostic challenge of a giant para-ovarian cyst in an adolescent girl in a low resource setting- a case report. Int J Surg Case Rep.

[8] Almahmeed E, Alshaibani A, Alhamad H, Abualsel A (2022). Giant paratubal cyst mimicking mesenteric cyst. Case Rep Surg.

[9] Vagholkar K (2025). Giant para tubal cyst in an adolescent girl mimicking a mesenteric cyst. Acta Med Bulg.

[10] Singh S, Agarwal I, Begum J, Bhardwaj B (2023). The burden of paraovarian cysts - a case series and review of the literature. Prz Menopauzalny.

[11] Bhansakarya R, Subedi S (2020). Laparoscopic management of large right paratubal cyst: a case report. JNMA J Nepal Med Assoc.

[12] Butureanu TA, Apetrei AM, Pavaleanu I, Haliciu AM, Socolov R, Balan R (2025). Extremely rare case of a giant paratubal cyst, coexisting with a mucinous cystadenoma, surgically treated through laparoscopy-a case report and review of the literature. Reports (MDPI).

[13] Archer S, Alaniz VI, Huguelet PS (2023). Surgical management of a giant paratubal cyst: a case report and review of the literature. Ann Pediatr Surg.

